# Bayesian spatio-temporal disease mapping of COVID-19 cases in Bangladesh

**DOI:** 10.1371/journal.pone.0316621

**Published:** 2025-02-14

**Authors:** Sefat - E- Barket, Md. Rezaul Karim, Md. Sifat Ar Salan

**Affiliations:** Department of Statistics and Data Science, Jahangirnagar University, Dhaka, Bangladesh; King Faisal University, SAUDI ARABIA

## Abstract

**Background:**

COVID-19 is a highly transmittable respiratory illness induced by SARS-CoV-2, a novel coronavirus. The spatio-temporal analysis considers interactions between space and time is essential for understanding the virus’s transmission pattern and developing efficient mitigation strategies.

**Objective:**

This study explicitly examines how meteorological, demographic, and vaccination with all doses of risk factors are interrelated with COVID-19’s complex evolution and dynamics in 64 Bangladeshi districts over space and time.

**Methods:**

The study employed Bayesian spatio-temporal Poisson modeling to determine the most suitable model, including linear trend, analysis of variance (ANOVA), separable models, and Poisson temporal model for spatiotemporal effects. The study employed the Deviance Information Criterion (DIC) and Watanabe-Akaike information criterion (WAIC) for model selection. The Markov Chain Monte Carlo approach also provided information regarding both prior and posterior realizations.

**Results:**

The results of our study indicate that the spatio-temporal Poisson ANOVA model outperformed all other models when considering various criteria for model selection and validation. This finding underscores the significant relationship between spatial and temporal variations and the number of cases. Additionally, our analysis reveals that maximum temperature does not appear to have a significant association with infected cases. On the other hand, factors such as humidity (%), population density, urban population, aging index, literacy rate (%), households with internet users (%), and complete vaccination coverage all play vital roles in correlating with the number of affected cases in Bangladesh.

**Conclusions:**

The research has demonstrated that demographic, meteorological, and vaccination variables possess significant potential to be associated with COVID-19-affected cases in Bangladesh. These data show that there are interconnections between space and time, which shows how important it is to use integrated modeling in pandemic management. An assessment of the risks particular to an area allows government agencies and communities to concentrate their efforts to mitigate those risks.

## Introduction

The year 2020 marked a pivotal moment in history when the COVID-19 pandemic swept across the globe, ushering in a crisis of unparalleled magnitude that left no country untouched. The disease in question is a highly transmissible human illness, which poses a novel challenge to the healthcare system due to its rapid infection rate and ease of transmission among individuals [1]. It usually takes 5 to 6 days for COVID-19 to show symptoms, but it can take anytime from 2 to 14 days [2]. During this long period, people infected with COVID-19 can give the virus to others around them without showing any symptoms [3]. However, while the virus knows no borders, its impact is far from uniform, exhibiting significant intensity and temporal progression disparities across diverse regions.

Spatial analysis primarily focuses on examining variations in space. The spatio-temporal analysis considers the interactions in space and, as a result, reveals the potential routes that the disease could take over time [4]. This integration identifies illness patterns that persist over time and extend across different spatial units [5]. Applying spatio-temporal modeling and mapping are significant and comprehensive techniques for fully understanding the dynamic nature of this global health issue and effectively responding to it within the context of the COVID-19 pandemic [6]. Through integrating spatial and temporal dimensions, spatio-temporal analysis has yielded significant insights into disease transmission monitoring and prediction modeling, ultimately contributing to developing a more deliberate and focused pandemic response. COVID-19 infections can exhibit geographical and spatio-temporal fluctuations due to the intricate interplay of various factors, including socioeconomic vulnerability, increase in population density, urbanization, and meteorological variables [7]. There is a potential for the observed effects to not be applicable at a local or individual level, leading to the occurrence of an ecological fallacy [8,9].

According to [10], infectious diseases like respiratory influenza, pneumonia, and respiratory syncytial virus change over time, as do the social, economic, and environmental conditions linked to them. These diseases include vector-borne ones like malaria, dengue, and Zika and sexually transmitted ones like HIV and syphilis. Therefore, because COVID-19 is an infectious circumstance, there is a good chance that it will spread spatially in a pattern that can be described. Much study has been done on the spatial and temporal patterns of COVID-19 using different modeling methods [11–14]. Shaopei [15] suggests a versatile Bayesian hierarchical framework that utilizes spatiotemporally changing coefficients to consider diversity in data rather than treating the covariate effects as constant. It is shown that for the COVID‐19 Delta variant cases, the eight considered environmental, sociodemographic, and public intervention factors are selected in different clusters, and the factor effects have distinct temporal behaviors depending on the groups. Researchers used the Mann-Kendall (M-K) and Pettit non-parametric tests to investigate spatiotemporal trends, and they detected cluster patterns in Bangladesh, a developing country, to track COVID-19 utilizing twelve essential disease-related spatial variables. Furthermore, the study found that COVID-19 confirmed cases are more common in highly populated urban areas and highly densely populated places where options for better living are abundant and also service facilities like transportation, healthcare, and daily consumables are conveniently accessible [16]. To date, there has been a lack of studies investigating the association of several potential risk variables, such as demographic characteristics, meteorological conditions, and complete vaccination coverage, with the occurrence of confirmed cases. Furthermore, none of the 64 districts of Bangladesh have had any research using Bayesian hierarchical spatio-temporal approaches to investigate the virus’s dynamic characteristics, which are constantly changing over space and time and are related to these risk factors, which could be vital in managing the ever-changing pandemic situation.

With the issues above in mind, the primary aim of this research is to investigate the implications of environmental and demographic factors, as well as the association of vaccination at all dosages, on the progression of the pandemic. By employing spatio-temporal models, this study investigates and presents findings on the substantial relationship of covariates to the occurrence of COVID-19 in the contiguous districts of Bangladesh, a nation severely affected by the global pandemic. To construct a spatio-temporal dataset covering 19 months, we combine information on reported instances of the disease with weather and vaccination data. This dataset is then combined with district-level demographic information to perform additional spatio-temporal analysis using those variables. A comprehensive discussion of the materials and methods employed, encompassing data sources and statistical models, is provided in section 2. In section 3, comprehensive justifications for the findings are covered. Section 4 provides an informative analysis of the concluding statements, accompanied by concise discussions.

## Materials and methods

### Data source and description

The daily affected cases data from June 5, 2021, to December 31, 2022, were collected from the Directorate General of Health Service (DGHS). Census information was collected from the Bangladesh Bureau of Statistics (BBS) in 2011. Based on a 2019 survey, data on household internet users (*%* ) has been collected from Pragatir Pathey Bangladesh (PPB). Meteorological data from the Bangladesh Meteorological Department (BMD) have been taken throughout the same period as the affected cases. To enhance the depth of our research, we introduced a new covariate related to vaccination. This covariate accounts for the vaccination status, including all doses administered, and it is based on data obtained from the COVID-19 Vaccination Dashboard for Bangladesh (VBD) during the same period. The description of the data used in this investigation is given in Table 1. We collected weekly average data on maximum temperature ( ℃ ) and humidity (*%*), alongside weekly aggregated data on vaccinations. In addition, the spatial domain consisted of districts (second administrative level) with 64 unique district names, while the temporal domain consisted of 83 weeks for consecutive districts. It is important to note that any personal information of individuals was not included in the dataset.

**Table 1:** Description of the study’s data sources.

Data	Min	Max	Mean	S.D	Source
Affected Cases	19108.97	68467.24	2221	556212	DGHS
Maximum Temperature	30.62	33.05	31.93	0.53	BMD
Humidity	74.39	86.25	80.91	2.48	BMD
Population Density	87	8229	1124.39	1057.13	BBS
Urban population	524572.79	1183705.46	83393	9317043	BBS
Literacy Rate (%)	34.98	76.51	52.56	9.76	BBS
Aging Index	20776	562276	168947.17	101038.71	BBS
Household Internet Users (%)	5	61.8	34.56	13.12	PPB
Vaccination (All Doses)	1221	386803	14783.016	47595.42431	VBD

### Criteria for variable selection

In the initial phase of our analysis, we incorporated various demographic variables, namely population density, urban population, total population, literacy rate, aging index, literacy rate, industrial population, and household internet users. However, the models were not well fitted, and for refinement, we introduced meteorological and vaccination with all dose variables into the equation. We employed the backward stepwise regression approach to enhance our models’ precision. The backward method systematically eliminated variables that did not contribute significantly to the models while retaining those that exhibited statistical significance.

### Transformation of variables

We used the logarithmic transformation method because the variables had different types of units, such as raw values and percentages. A logarithmic scale was used to transform the values of the vaccination variable and some demographic variables, such as urban population, population density, and the aging index. The transformed values were used instead of the original ones for the Bayesian hierarchical model.

### Spatio-temporal random effects for data from arial units

In the Bayesian spatio-temporal framework, when creating a prior distribution for spatially autocorrelated random variables, a typical strategy is to combine the intrinsic conditional autoregressive (ICAR) distribution with a uniform prior for the intercept that ranges from negative to positive infinity [6]. If the joint posterior distribution is accurate which is gained from the joint prior distribution function, this does not present a challenge for Bayesian modeling [17]. A notable change to conditional autoregressive (CAR) models was provided by [18] to prevent singularity. Their suggestion involves using the equation


$$Q\left(W,\rho \right)=\rho \left(D_{w}-W\right)+\left(1-\rho \right)I$$

where *I* is a representation of the identity matrix with dimensions NXN, and 1 is a vector of ones with dimensions NX1. The parameter known as *ρ*, which regulates the degree to which spatial correlation is present, can include any value between 0 and 1. While putting ρ=1*,* the modified equation turns back to the original specification. In this case, the element in the neighboring nth dimensional matrix W that corresponds to the ith row and jth column is denoted by $\omega_{ij}$. The spatial variance of the random effects is $\tau^{2}$and *ρ*.

The outcome of this leads to a CAR distribution.


$$\psi_{i}|\rho ,\tau^{2},\psi_{j},j\neq i\sim N\left(\frac{\rho \sum_{j}\omega_{ij}\psi_{j}}{\rho \sum_{j}\omega_{ij}+1-\rho },\frac{\tau^{2}}{\rho \sum_{j}\omega_{ij}+1-\rho }\right)$$ (1)

and this prior distribution of the CAR model is referred to as $NCAR\left(\psi \text{|}\rho ,\tau^{2},W\right)$.

For this study, the response variable $Y_{it}$ follows a Poisson distribution. This distribution reflects the confirmed number of cases in a country where *i* indicates districts i=1,2,3,…,64 in month $t\left(t=1,2,3,\ldots ,83\right)$ and $n_{it}$ be the population size at district *i* during that time *t*. The design matrix *X* denotes the covariates, while *β* represents a vector containing the fixed effects parameters associated with these covariates.


$$log\left(\mu_{it}\right)=\eta_{it}=X\beta +log\left(n_{it}\right)$$ (2)

Adding the random spatial-temporal effect $\psi_{it}$ to Equation 2 yields Equation 3 which is


$$log\left(\mu_{it}\right)=\eta_{it}+\psi_{it}$$ (3)

It is necessary to decompose the spatio-temporal random effect $\left(\psi_{it}\right)$ to capture its many spatio-temporal properties, such as spatio-temporal interactions. This is because the spatio-temporal random effect has multiple aspects due to the multiple aspects of the spatio-temporal random effect [6].

#### Spatio-temporal Poisson linear trend model (SPLTM).

A spatio-temporal Poisson model that is assumed to be appropriate to investigate the presence of any simple linear trend in the random effects $\psi_{it}$ for each district *i* is given by


$$\psi_{it}=\beta_{1}+\phi_{i}+\left(\beta_{2}+\delta_{i}\right)\frac{t-\bar{t}}{T}$$

where $\bar{t}=\frac{T+1}{2}$*.* In a Bayesian model, the parameters known as $\beta_{1}$ and $\beta_{2}$ represent the overall intercept and slope (trend), respectively, and will be assigned a uniform prior distribution. The parameters $\phi_{i}$ and $\delta_{i}$ represent the incremental intercept and slope parameters for the $i^{th}$ districts, where *i* ranges from 1 to 64. These parameters are allocated in a conditional autoregressive (CAR) prior distribution in Equation 1, with distinct values of *ρ* and $\tau^{2}$. Specifically, the parameters $\phi =\left(\phi_{1},\ldots ,\phi_{64}\right)$ and $\delta =\left(\delta_{1},\ldots ,\delta_{64}\right)$ follow the CAR prior distribution that is $\phi ∼NCAR\left(\phi \text{|}\rho_{int},\tau_{int}^{2},W\right)$ and $\delta ∼NCAR\left(\delta \text{|}\rho_{slo},\tau_{slo}^{2},W\right)$ respectively. The variables $\rho_{int}$ and $\rho_{slo}$ are assigned separate prior distributions that follow a uniform distribution inside the interval (0, 1). This indicates that their values can vary between 0 and 1. Additionally, the variance parameters, $\tau_{int}^{2}$*,* $\tau_{slo}^{2}$ are assumed to follow an inverse gamma prior distribution. Finally, putting the random effect, $\psi_{it}$ into Equation 3 we get the final model as


$$log\left(\mu_{it}\right)=\eta_{it}+\beta_{1}+\phi_{i}+\left(\beta_{2}+\delta_{i}\right)\frac{t-\bar{t}}{T}$$

#### Spatio-temporal Poisson ANOVA model (SPAM).

[19] put out a model in which the assumptions of linearity do not have to be fulfilled. The model can be interpreted as a temporal counterpart of the spatial structure element. However, this model considers the additive effects of space and time; it does not consider the interactions. [20] developed a method for analyzing data that considers the interactions between spatial and temporal dimensions. This method uses the Analysis of Variance technique to construct a model considering spatial and temporal significant effects. Equation 4 represents the spatial and temporal effect of interaction.


$$\psi_{it}=\phi_{i}+\delta_{t}+\gamma_{it}\;;\;i=1,2,3,\ldots ,64,\;t=1,2,3,\ldots ,83$$ (4)

Considered as random effects, each of the three parameter sets in Equation 4 have its own probability distribution given by:


$$\phi |\rho_{s},{\tau_{S}}^{2},W\sim NCAR(\phi |\rho_{s},{\tau_{S}}^{2},W)\delta |\rho_{T},{\tau_{T}}^{2},D\sim NCAR(\delta |\rho_{T},{\tau_{T}}^{2},D)\gamma_{it}\sim N\left(0,{\tau_{I}}^{2}\right),i=1,2,3,\ldots ,64,t=1,2,3,\ldots ,83$$

where the temporal adjacency matrix TXT is denoted by the symbol *D*. In this matrix, $d_{ij}$ has the value one if the absolute difference between *i* and *j* is 1, and it has the value 0 in any other case. The prior distributions for the parameters $\rho_{S}$ and $\rho_{T}$ are each given their independent uniform distribution in the unit interval (0,1). However, the prior distributions for the variance parameters $\tau_{S}^{2},\tau_{T}^{2}$, and $\tau_{I}^{2}$ are given as inverse gamma distributions. Following the dissection of the spatio-temporal random effect and its incorporation into the linear predictor $\eta_{it}$, as outlined in Equation 3, the results were as follows:


$$\log\left(\mu_{it}\right)=\eta_{it}+\phi_{i}+\delta_{t}+\gamma_{it}$$

#### Spatio-temporal Poisson separable model (STSM).

An additional possibility is the separable model, which provides an overall temporal trend and temporally specific geographical impacts. This model is an alternative to the spatio-temporal Poisson ANOVA model (SPAM) [6]. In this scenario, the spatial effects $\phi =\left(\phi_{1t},\phi_{2t},\ldots ,\phi_{64t}\right)$ for one by one t=1,…,83 and also for others $\delta =\left(\delta_{1},\ldots ,\delta_{83}\right)$ are attributed to normal conditional autoregressive (NCAR) models are shown in the following Equation 5.


$$\psi_{it}=\phi_{it}+\delta_{t}\;;\;i=1,2,3,\ldots ,64,\;t=1,2,3,\ldots ,83$$ (5)

$$\phi_{t}\left|\rho_{s},{\tau_{t}}^{2},W\sim NCAR\left(\phi \text{|}\rho_{s},{\tau_{t}}^{2},W\right),t=1,2,3,\ldots ,83\delta \right|\rho_{T},\tau^{2},D\sim NCAR(\delta |\rho_{T},\tau^{2},D)$$

where details about *D* is discussed above. The prior distributions for the parameters $\rho_{T}$ and $\rho_{S}$ are each given their independent uniform distribution within the unit interval 0 and 1, but the prior distributions for the parameters of variance $\tau_{t}^{2},t=1,2,\ldots ,83$ and $\tau^{2}$ are given as inverse gamma distributions. After the decomposition of the spatio-temporal random effect $\psi_{it}$ and its addition to the linear predictor $\eta_{it}$ as described in Equation 3, the model accurately considers spatial and temporal dependencies, enhancing prediction accuracy and reliability.


$$\log\left(\mu_{it}\right)=\eta_{it}+\phi_{it}+\delta_{t}$$

#### Poisson temporal model for spatiotemporal effect (TMS).

A hypothesis is made that a particular scenario within the framework of the separable model, as discussed above, corresponds to a temporal autoregressive model with a lag of one. The simplification that results from the fact that the parameter $\delta_{t}$ has a value of zero for all *t* values with the notation that $\delta_{t}=0$ and


$$\psi_{it}=\phi_{it}$$

$$\phi_{t}\left|\phi_{t-1},W\sim N\left(\rho_{T,1}\phi_{t-1},\tau^{2}Q{\left(W,\rho_{S}\right)}^{-1}\right),t=2,3,\ldots ,83\phi_{t}\right|W\sim N\left(0,\tau^{2}Q{\left(W,\rho_{S}\right)}^{-1}\right),$$

In addition to this, an autoregressive of order 2 can be written by including an additional term $\rho_{T}{,}_{2}\phi_{t-2}$ [17]. After breaking down the random effect and adding in their respective components to Equation 3, the equation is as follows:


$$log\left(\mu_{it}\right)=\eta_{it}+\phi_{it}$$

### Computational procedure

The matrix scatter plot of the risk factors was displayed using the GGally packages in RStudio. Map visualization requires shapefiles, a component of the second administrative layer, which was utilized using the readShapePoly function. The poly2nb suggests shared boundary sites by compiling a list of adjacent districts based on shared boundaries. By adding spatial weights, the nb2listw function enhances an already-existing neighbors list.

The ST.CARanova, ST.CARsepspatial, and ST.CARar models have been incorporated into the CarBayesST [21] package to represent all of the spatio-temporal models. All of these models operate using the argument package=CARBayesST within the Bcartime function in the bmstdr package [17]. All the spatio-temporal models were acquired by only changing the arguments in the Bcartime function. We obtain the output of the models by putting “linear”, “anova”, “sepspatial”, and “ar” (by default creates TMS(1), and by adding additional argument AR = 2, we can get TMS (2)), correspondingly. The doBy package assesses model validation criteria and determines which model yields the lowest root mean squared error (RMSE). The models were executed for 120,000 iterations, with the first 20,000 iterations discarded as burn-in. Under the Markov Chain Monte Carlo (MCMC) framework, the data were saved following a thinning process of 10 iterations to reduce autocorrelation. An additional argument, validrows, is incorporated into the model validation process.

### Ethical statement

Not applicable.

## Results

**Fig 1:**
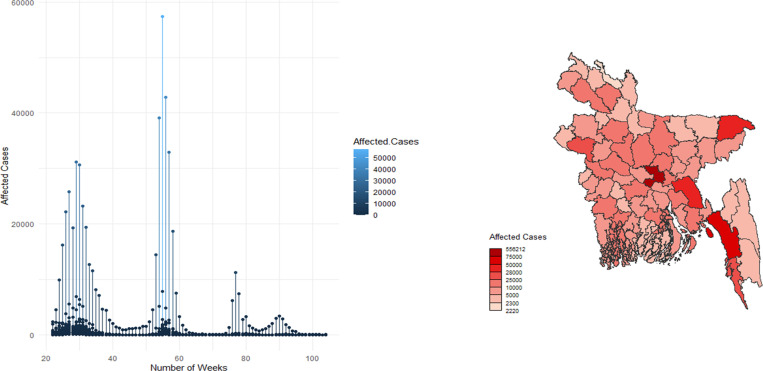
Weekly report of COVID-19 cases and spatial distribution across 64 districts of Bangladesh. (A) The number of COVID-19 cases reported each week between June 5, 2021 and March 31, 2022; (B) District-by-district breakdown of affected cases across the country.

(Fig 1) illustrates the fluctuation of affected cases with the ongoing week numbers. Initially, the temporal risk appeared to fluctuate during weeks 22 through 30. The $29^{th}$ and $30^{th}$ weeks revealed a large number of reported instances. Infected cases have been found consistently from the $40^{th}$ to the $50^{th}$ week (October to December) of 2021. The highest temporal risk is in the winter (January) of 2022. In the $55^{th}$ week, the country met up with the highest total affected cases, surpassing almost 58,000. In Fig 1 Dhaka marks the highest number of affected cases showing by darkest red color. Meanwhile, Lalmonirhat shows up as having the lowest affected cases marked by the lightest red color. Khagrachari and Bandarban, these two hilly districts, tend to have lower affected cases as the population density and urban population are low here.

**Fig 2:**
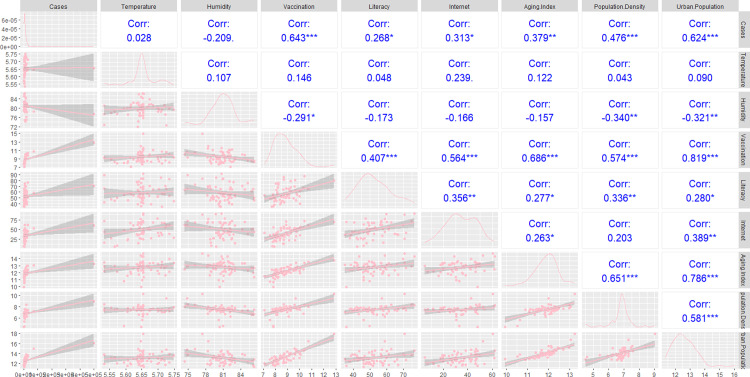
A scatter plot of the reported cases and associated chosen factors in pairs. *** refers *p*-value <  0.001, ** refers *p*-value <  0.05 and *  refers *p*-value <  0.1.

**Fig 3:**
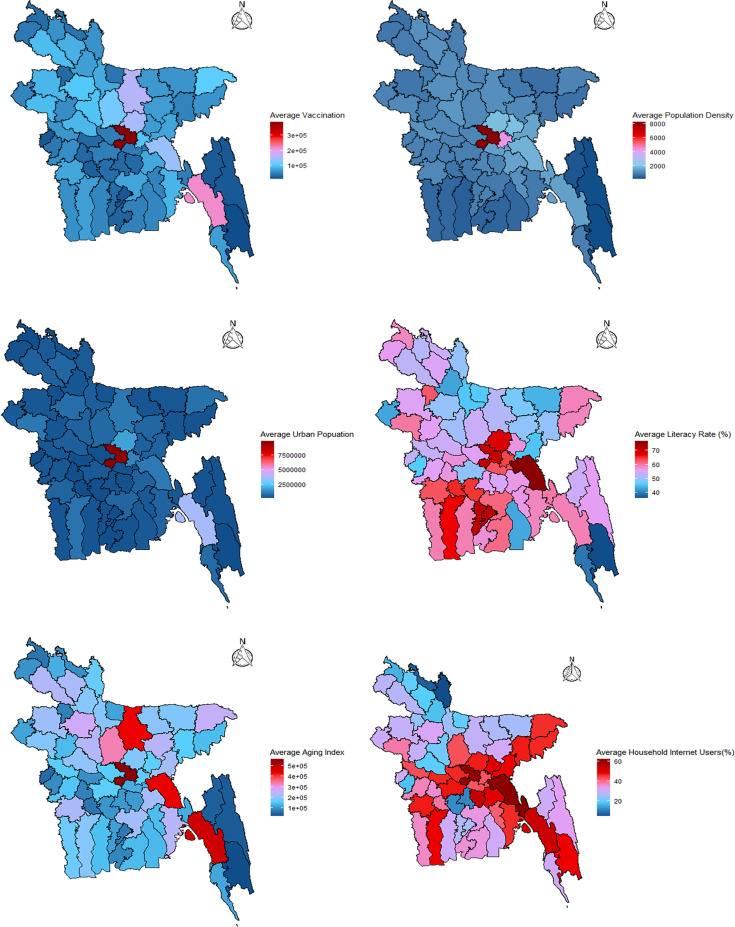
District-wise variation in (A) vaccination, (B) population density, (C) urban population, (D) literacy rate, (E) aging index, and (F) internet users.

The scatter plots showing the response and variables plotted in pairs are presented in (Fig 2). The estimations of the variables’ kernel densities are presented in this plot via the diagonal panels. The first column demonstrates no correlation between meteorological variables and the number of cases. Three covariates, namely population density, urban population, and vaccination, each exhibited a significant and moderately positive correlation with the variable of interest. Literacy rate, aging index, and households with internet access point to having weak relationships with affected cases.

(Fig 3) illustrates the characteristics of the relevant covariates about the spatial domain. A notable disparity is seen among the various districts of Bangladesh, as indicated by the observed variations in all the numbers. Dhaka, the capital city of Bangladesh, exhibits the highest population density.

and urban population, as expected for a city situated in one of the most populous countries globally. The distribution of vaccinations is determined by the population size of individual districts, resulting in Dhaka district residents receiving the highest vaccine doses. The population density in the hilly districts of Khagrachari, Bandarban, and Rangamati is relatively low. Vaccination coverage is also significantly low in these districts. The district with the highest rate of literacy is Cumilla. Bandarban lags in terms of educational opportunities due to a lack of vital facilities required for education. Dhaka, Cumilla, and Feni districts typically have a large percentage of people using the internet at home, whereas the number of people using the internet at home in Kurigram is still relatively low.

**Fig 4:**
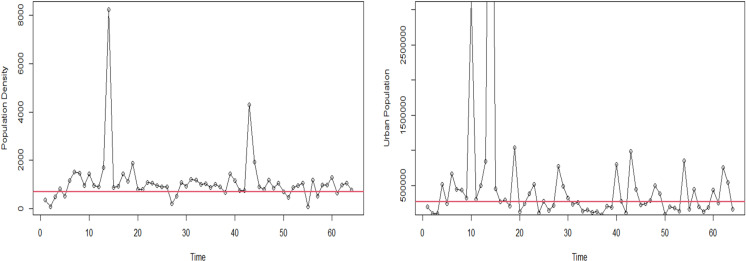
Line graph showing threshold point of (A) population density and (B) urban population. The estimated value of *α* in the SPLTM model shown in Table 2, which captures the overall trend of COVID-19 cases throughout 64 districts of Bangladesh, exhibits a statistically significant increase. Nevertheless, it is essential to note that considerable differences exist in the levels of trends seen across different districts. This is evident from the trend variation level of 5.136, accompanied by a reasonable interval ranging from 3.208 to 8.806. The data suggests a weak association between the observed trends, with a coefficient of 0.098. Remarkably, despite the lack of a strong association, the model that assesses spatio-temporal variation every week exhibits the most significant value for the DIC and WAIC among all the models (see Table 3).

Bangladesh’s different-sized districts are accountable for the differences in the number of people living in cities and the density of those people. This study aims to find districts where COVID-19 outbreaks are more likely to happen. Then, it might make sense to group them by population density and urbanization. For the COVID-19 pandemic to spread quickly and easily, many people must simultaneously be in the same place. Two main factors determine how the pandemic changes: the number of people living in cities and the population density. These variables have been split in half to make the analysis more accessible. After looking at (Fig 4), a level of 700 is an excellent place to start separating the population density into two groups. A population density of fewer than 700 people is called “low population density”, while a population density of more than 700 people is called “high population density”. It can be seen in (Fig 4) that most of the densities are higher than 700 in 53 of the 64 districts. A visual look at the data showed that most districts have population densities of more than 700 people per square kilometer, which means that districts with a population density above this level have a higher concentration of people living there than the other districts. The median value of 267048.5 is also used as a cutoff point for the urban population. This value divides the population into two groups based on urban population.

**Table 2:** The parameter estimate for spatio-temporal models that elucidate the COVID-19 affected case factors in Bangladesh with a 95% credible.

STSM	-1.902 (-6.588, 3.853)	0.155 (-0.176, 0.19)	-0.008 (-0.016, 0.007)	-0.077 (-0.117, -0.048)	0.259 (0.192, 0.305)	0.045 (0.016, 0.079)	1.072 (1.026, 1.125)	1.069 (1.054, 1.082)	0.016 (-0.002, 0.030)	-1.264 (-1.593, -1.011)	-0.039 (-0.044, -0.027)						0.959 (0.426, 1.889)				0.003 (0.000, 0.010)	0.095 (0.002, 0.330)		
TMS(2)	18.488 (17.924, 19.313)	-0.006 (-0.017, 0.002)	-0.002 (-0.005, 0.004)	-0.256 (-0.274, -0.246)	0.174 (0.156, 0.220)	0.037 (0.015, 0.072)	1.303 (1.281, 1.314)	0.971 (0.956, 0.984)	-0.046 (-0.048, -0.043)	-2.649 (-2.740, -2.583)	-0.065 (-0.069, -0.062)				29.933 (25.973, 33.345)						0.661 (0.597, 0.723)			
TMS(1)	19.181 (16.854, 21.298)	-0.179 (-0.188, -0.169)	0.039 (0.028, 0.050)	-0.215 (-0.240, -0.197)	0.216 (0.193, 0.246)	0.049 (0.034, 0.066)	1.306 (1.282, 1.348)	0.984 (0.971, 0.993)	-0.047 (-0.051, -0.039)	-2.602 (-2.692, -2.527)	-0.059 (-0.064, -0.055)				32.291 (27.596, 38.670)						0.631 (0.554, 0.714)	0.002 (0.000, 0.008)	-0.032 (-0.069, 0.003)	0.368 (0.322, 0.413)
SPAM	28.736 (26.666, 30.288)	-0.114 (-0.156, 0.214)	-0.006 (-0.008, -0.003) *	-0.269 (-0.278, -0.259) *	0.162 (0.138, 0.195) *	0.013 (0.004, 0.035) *	1.438 (1.425, 1.448) *	0.949 (0.942, 0.954) *	-0.05 (-0.054, -0.046) *	-3.240 (-3.378, -3.071) *	-0.077 (-0.080,-0.075) *					10.294 (6.713, 16.024)	11.379 (7.337, 17.806)	31.389 (29.219, 33.358)			0.050 (0.002, 0.195)	0.278 (0.025, 0.642)		
SPLTM	-9.955 (-10.046, -9.877)	-0.045 (-0.046, -0.044)	0.044 (0.043, 0.044)	-0.194 (-0.196, -0.193)	0.127 (0.121, 0.133)	0.144 (0.139, 0.148)	1.075 (1.065, 1.091)	1.036 (1.027, 1.052)	0.009 (0.009, 0.009)	-0.839 (-0.859, -0.824)	0.010 (0.010, 0.011)	0.208 (0.197, 0.220)	0.133 (0.082, 0.229)	5.136 (3.208, 8.806)					0.077 (0.002, 0.271)	0.098 (0.004, 0.320)				
Characteristics	Intercept	Temperature	Humidity	Vaccination (All Doses)	Low Population Density	High Population Density	Low Urban Population	High Urban Population	Literacy Rate (%)	Aging index	Houses with Internet (%)	$\hat{\alpha }$	$\hat{{\tau^{2}}_{int}}$	$\hat{{\tau^{2}}_{slo}}$	$\hat{\tau^{2}}$	$\hat{{\tau^{2}}_{S}}$	$\hat{{\tau^{2}}_{T}}$	$\hat{{\tau^{2}}_{I}}$	$\hat{{\rho^{2}}_{int}}$	$\hat{{\rho^{2}}_{slo}}$	$\hat{\rho_{S}}$	$\hat{\rho_{T}}$	$\hat{\rho_{1}}$	$\hat{\rho_{2}}$

**Table 3:** Model selection criteria for five spatio-temporal Poisson models. The penalties for DIC and WAIC are represented, respectively, by pD and.pW.

Models	DIC	pD	WAIC	pW	LMPL	Loglikelihood
SPLTM	29665.726	3862.944	29280.772	2954.829	−23226.430	−7602.921
SPAM	**20531.136**	2786.620	**19995.462**	1677.445	**−11296.654**	**−7527.795**
TMS (1)	20741.633	2829.332	20410.810	1725.014	−11583.073	−7537.948
TMS (2)	20671.844	2781.280	20201.637	1620.617	−11504.481	−7554.642
STSM	26086.605	3463.752	25650.825	2246.816	−18278.866	−7579.551

Table 2 demonstrates that the aging index has a statistically significant negative interrelation with infected cases, with a coefficient of exp(−3.240) and a 95% credible interval of exp(−3.378, −3.071). This indicates a reduction of around 7.97% in the infected cases for every 1% rise in the aging index. Another factor that has a negative and statistically significant association with affected cases is the number of internet users in the household, with a coefficient of exp(−0.077) and a 95% credible interval of exp(−0.080, −0.075) indicating that for every unit increase in the number of internet users, there is a decrease in the affected cases by roughly 94.08%.

Both urban population categories have statistically significant 95% credible intervals with positive coefficients. The coefficient value of exp(1.438), with intervals exp(1.425) and exp(1.448), indicates that a lower urban population has a positive relationship with the rise of infected cases. Similarly, it can be observed that there is a significant association between the number of COVID-19 cases and high urban populations, as indicated by a coefficient of exp(0.949) with 95% credible intervals exp(0.942, 0.954). The association of population density with the rise of COVID-19 infection cases is widely acknowledged, as evidenced by the statistically significant relationship between these two variables, supported by a 95% credible interval. The variable representing low population density exhibits a positive coefficient of exp(0.162), with statistically significant credible intervals ranging from exp(0.138) to exp(0.195). Additionally, it is observed that there is a positive and statistically significant relationship between the highly dense population and the number of infected patients. The coefficient value for this relationship is exp(0.013), with a 95% credible interval exp(0.004, 0.035). Both categorizations ultimately demonstrate a favorable correlation with the growth of the pandemic.

In addition, humidity is a covariate that has substantial adverse effects on confirmed cases, with coefficients of exp(−0.006) with 95% credible intervals exp(−0.008, −0.003). These associations were found to be statistically significant. This indicates that a rise in COVID-19-affected patients negatively correlates with increased humidity. Vaccinations have a significant relationship with the decline of the pandemic, as demonstrated by all proposed models. With a coefficient of exp(−0.269) and credible intervals of exp(−0.278, −0.259), it can be shown that for every 1% increase in vaccination of all doses, there will be an approximately 87.19% decrease in the number of instances of virus. Based on the spatio-temporal Poisson ANOVA model, the rise of infected cases will be reduced by 95.69% for every one-unit literacy rate increase, which goes up roughly by exp(−0.050). The temperature did not show any significant association with the rising or decreasing COVID-19-infected cases, which is consistent with the paired scatter plot after analyzing the association of temperature on infected cases from 95% credible intervals.

According to the findings presented in Table 2, the estimation for the spatial correlation parameter $\rho_{S}$ is 0.050, accompanied by a 95% credible interval ranging from 0.002 to 0.195, signifying the existence of a weak positive spatial correlation. A positive spatial correlation of low magnitude suggests a certain level of connection between the values of a variable in adjacent or proximate spatial units (such as geographical places) but with a comparatively limited degree of strength.

Conversely, the temporal autocorrelation coefficient $\rho_{T}$ is 0.278, with a 95% credible interval of (0.025, 0.642), higher than the spatial correlation coefficient. This indicates a weak positive temporal correlation between observations at different time intervals, a small but significant in terms of spatial correlation. Furthermore, the estimated spatial variation resulted in a value of 10.294, which means that the variables’ values exhibit spatial heterogeneity, indicating that they are inconsistent across the entire district but vary from one district to another. Also, the estimated temporal variation was found to be 11.379.

Table 3 presents the MCMC sample used to construct the model choice criterion. The spatio-temporal Poisson ANOVA model (SPAM) is the one that fits the data best, according to both the lowest DIC and WAIC. The LMPL value of −11296.654 is substantially higher, which indicates that it performs more accurately as a forecasting tool. The value of −7527.795 for Loglikelihood implies a model better fitting to the data. Furthermore, to examine model validation, only four models are examined, excluding the STSM because fitting this model does not currently take missing values (see Table 4) [17]. Refitting the SPAM model by excluding 10% of arbitrarily selected data rows at this point. The SPAM model has the lowest RMSE, MAE, CRPS, and CVG values compared to others. Findings in Table 4 show that the SPAM model has the lowest RMSE, which implies that this model minimizes the typical errors in prediction. It also has the lowest MAE and CRPS values, which indicate that it provides accurate probabilistic forecasts. In addition, the SPAM model has the highest CVG, which indicates that a higher percentage of actual data points fall inside its prediction intervals, indicating that coverage is likely to be satisfactory with this model.

**Table 4:** Model validation statistics for the four models.

Models	RMSE	MAE	CRPS	CVG
SPLTM	6.18	3.15	2.55	94.09%
SPAM	**4.91**	**2.48**	**1.56**	**95.52%**
TMS(1)	5.64	2.99	2.24	94.21%
TMS(2)	5.03	2.56	1.67	94.94%

**Fig 5:**
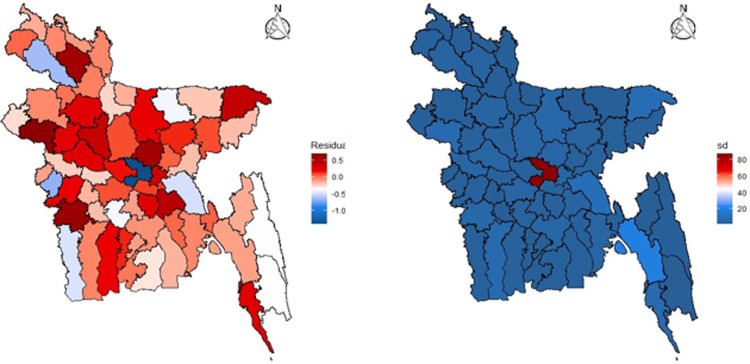
Residual plot by district, (A) spatially aggregated residuals and (B) residual standard error.

During the MCMC iteration, fitted values are computed for each observed response. Subsequently, the residuals are calculated, aggregating these values based on the temporal domain’s range. This aggregation process yields spatial residuals and provides us with the standard deviation of residuals for each specific spatial domain, referred to as *i*. Spatially aggregated residuals from the SPAM model in (Fig 5) do not visualize any systematic pattern that needs further analysis. Sometimes, spatially aggregated residuals do not seem to reveal the patterns in the data.

**Fig 6:**
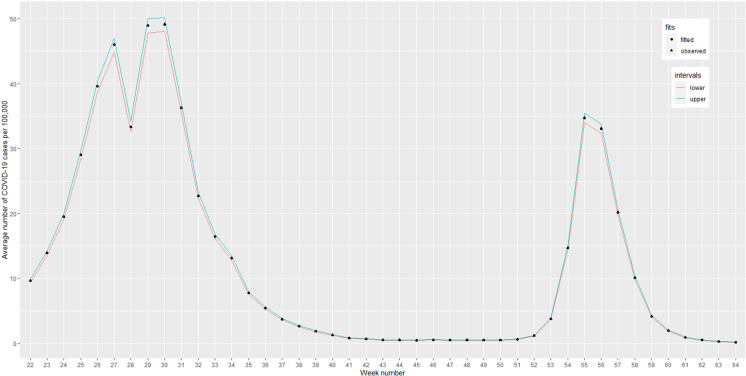
Time-series plot with 95% confidence intervals for observed and fitted COVID-19 cases.

However, the residual standard error provides a solution, allowing us to pinpoint geographical districts where the model performs well and districts where it does not. By mapping the residual standard error, we can present the model’s performance across the districts clearly and intuitively. A map showing uniform residual standard error suggests that the model predicts with similar accuracy and consistency across all the neighborhoods with effective generalization. Moreover, (Fig 5) demonstrates that the residual standard error from the SPAM model effectively accounts for the variation among districts, except Dhaka, highlighted in red. Since Dhaka is a megacity city with a very high population density, the number of confirmed cases is significantly larger than in other cities. An alternative method to assess the model’s fit to the data indicates a close alignment between the observed and fitted lines, signifying data-fitting solid performance. There is a minor overestimation at specific points, notably at week numbers 27, 29, 30, 55, and 56, as shown in (Fig 6). In addition, the upper and lower boundaries for the fitted values are pretty close to the fitted values, which suggests a very high degree of accuracy of the well-fitted spatio-temporal ANOVA model due to the enormous sample size of the spatio- temporal data set.

## Discussion

The findings underscore the gravity of the situation in Dhaka, which has the highest incidence of infected cases. Variations in the affected cases have been observed across different week numbers. Our study employed a Bayesian Poisson spatio-temporal framework to explore the relationship between COVID-19 infected cases with various covariates. We rigorously evaluated five distinct models using statistical criteria. Our analysis revealed that the SPAM model outperformed the other models, as evidenced by its lower DIC (20531.136) and WAIC (19995.462) values, indicating its superior fit to the data. The coefficients of the SPAM model revealed two significant factors: high and low urban populations and high and low population densities, both correlated with the number of COVID-19-affected cases. These findings provide valuable insights into the progression of the pandemic. The estimated spatial correlation suggests the presence of both weak and positive spatial correlation. However, the value of the temporal coefficient is higher than the spatial correlation, indicating a small but significant effect. Additionally, the estimated spatial variation demonstrates spatial heterogeneity, indicating disparities across the district but variation between districts. The validation results in Table 4 for the SPAM model further support the importance of considering spatio-temporal effects in explaining the variation in affected cases across Bangladesh.

In 2020, when the global SARS-CoV-2 pandemic began, there was interest in whether milder summer temperatures, like those witnessed in 2003, would reduce virus spread. The research discovered that temperatures from 4 to 40 degrees Celsius unaffected the virus’s spike protein integrity; however, spike protein denatured at 50 ℃ . Changing respiratory system temperatures did not affect SARS-CoV-2 particle-lung epithelial cell interactions [22]. Ultimately, according to the study, temperature did not substantially correlate with COVID-19 transmission and severity. The spatio-temporal Poisson ANOVA model identifies a significant negative association between the increasing number of affected cases and humidity. Despite having a population of 163 million people and more COVID-19 cases than the rest of the world, Bangladesh appears to have a less severe COVID-19 problem due to its location in the tropical monsoon zone. Even though this country has the highest population density and cannot be tested regularly, COVID-19 may spread slowly because of often severe humidity [23]. Furthermore, the study noted that a significant number of cases clustered in humidity levels ranging from 55% to 65%, however, we did not investigate the prevalence of such clusters in any humidity range throughout our study.

Again, significant connections between population density and urban population with the COVID-19 cases have been observed in this study. Bangladesh has the seventh highest population density in the world, with 1107 people living per square km [24]. Individuals residing in areas characterized by high population density are anticipated to experience a higher frequency of social meetings than those living in rural environments, making the virus transmission easier [25]. Additionally, limited space for physical distancing can be challenging, and infected individuals may struggle to isolate themselves, increasing the risk of the virus to spread. The urban population is associated with COVID-19 cases, evidenced by heightened travel and interaction resulting from increased population density. The reliance on public transportation further facilitates the virus to spread, and the diverse population introduces various virus strains, complicating control efforts.

The result discovered an adverse causal connection between the number of COVID-19 cases and the aging index. Contrary to the global trend, the virus is most deadly for those aged 21 to 40, with over half the fatalities (51.49%) occurring in those 60 and older [26]. A reason could be that as people age, their immune systems deteriorate, making them more susceptible to underlying health issues like COVID-19. One explanation could be that elderly individuals tend to spend less time outside their homes and travel shorter distances from where they dwell [27]. They are also less inclined to participate in large meetings or social events, whereas younger people tend to spend more time with others who are not family members [28].

To combat the pandemic, mass vaccination and herd immunity promotion through advertising have proven effective [29,30]. The Bangladesh Government began a vaccination campaign on January 27, 2021, targeting 80% of adults, initially prioritizing frontline workers and those aged 40 and above [26,31]. Vaccine distribution primarily happened in Dhaka’s tertiary healthcare centers, with additional doses allocated to district hospitals and Upazila health complexes [32]. Literate individuals make informed decisions about immunizations, seek proper medical advice, and better comprehend vaccination schedules, testing procedures, and quarantine instructions, limiting the cruciality of pandemics. Internet users have convenient access to COVID-19 safety information, and the growing availability of telemedicine and virtual health services promotes healthier habits and lowers infection risk by curbing the virus transmission.

## Conclusions

This study examines the temporal and spatial dynamics of COVID-19 and the relationship between vital factors such as demographics, climate, and vaccination with the virus-affected people. In light of model selection criteria and residual plots, we can conclude that the SPAM model outperforms other models and captures the spatio-temporal heterogeneity thoroughly in this study. This model, designed explicitly for spatial and temporal data, shows that the maximum temperature has no significant correlation with the impacted cases of the two climatic variables. Furthermore, population density and urban population have statistically significant and positive associations with the target variable, consistent with the findings of the pairwise scatterplot. Humidity, literacy rate, household internet users, immunization, and age index are all strongly associated with impacted cases, suggesting they may reduce COVID-19 cases. In this heterogeneous context, the COVID-19 pandemic is associated with spatial characteristics and spatio-temporal patterns that have implications for pandemic preparedness, as they enable the identification of hotspots, prediction of outbreaks, evaluation of interventions, the establishment of early warning systems, distribution of vaccines, and optimization of resource allocation and public health investments to better prepare for future pandemics. The results of this study can aid research institutes in planning future research and development.

## Study limitations

The study focuses solely on some specific demographic, meteorological, and vaccination variables. To better understand the dynamics of a pandemic, potential risk factors such as airborne chemical pollution, environmentally hazardous toxins, access to healthcare, income and employment, industrial population, and poverty should be analyzed at the district level, which may lead to the discovery of additional noteworthy information. Nevertheless, future research in multiscale spatio-temporal modeling is necessary as it simultaneously analyzes several spatial and temporal scales and integrates data of larger-scale and individual-level movements. This enables timely response by identifying risky hotspots before outbreaks spread to every corner of the country.

## Supporting information

S1 Data Raw data supporting the findings of this study. (CSV)
